# GAINING INTERPROFESSIONAL COMPETENCE THROUGH A GERIATRICS CASE CONFERENCE

**DOI:** 10.1093/geroni/igz038.543

**Published:** 2019-11-08

**Authors:** Amber S McIlwain, Danielle Backus, Kristine Marcus, Jeff Fortner

**Affiliations:** Pacific University, Hillsboro, Oregon, United States

## Abstract

There is increased demand to provide health professions students with interprofessional education and practice experience. Interprofessional Case Conferences (ICC) allow students to work in teams to learn about different professions while exploring a topic through the lens of an interprofessional core competency. The goal of this particular ICC was to provide students an experience to witness and discuss team-based, person-centered care for a common geriatric disorder. The case was designed to develop the interprofessional competency of teams and teamwork. Students were divided into teams and observed live vignettes of a care conference involving an 80-year old female admitted to a rehabilitation facility following a hip fracture and replacement. Students witnessed how patients, families, and healthcare providers work together during a stressful time. After each vignette, students discussed questions related to miscommunications, motivations of the different actors involved, and how the healthcare team should respond. In the first offering, 93 students participated, increasing to 150 in the next year. Students completed a post-survey to determine if the session delivered a positive interprofessional experience. The average positive response rate was 92.5% (92-94%, n = 53) in year-1 and 93.5% (90-97%, n = 71) in year-2. By allowing students to witness a simulated live care conference, they had a tangible event to dissect instead of discussing hypotheticals. By discussing a geriatrics case in a rehabilitation setting, students witnessed how numerous healthcare professions coordinate care for a patient and her family, thereby demonstrating competence in teamwork.

